# Degradation of Minocycline by the Adsorption–Catalysis Multifunctional PVDF–PVP–TiO_2_ Membrane: Degradation Kinetics, Photocatalytic Efficiency, and Toxicity of Products

**DOI:** 10.3390/ijerph182312339

**Published:** 2021-11-24

**Authors:** Chengzhi Zhou, Yanlong Sun, Fan Zhang, Yuandong Wu

**Affiliations:** 1 Qingdao Engineering Research Center for Rural Environment, College of Resource and Environment, Qingdao Agricultural University, Qingdao 266109, China; zhoucz@qau.edu.cn; 2 College of Geography and Environmental Sciences, Zhejiang Normal University, Jinhua 321004, China; sunyanlong@zjnu.edu.cn; 3 School of Electrical and Information Technology, Yunnan Minzu University, Kunming 650031, China; zhangfan2819@163.com; 4 Shenzhen Institute, Peking University, Shenzhen 518000, China

**Keywords:** electrospinning, minocycline, photocatalytic oxidation, PVDF–PVP–TiO_2_ fiber mats, degradation mechanism

## Abstract

The photocatalytic degradation of minocycline was studied by using polyvinylidene fluoride–polyvinylpyrrolidone–TiO_2_ (PVDF–PVP–TiO_2_) fiber mats prepared by an electrospinning technology. The influences of the TiO_2_ dosage, minocycline concentrations, inorganic anions, pH values, and dissolved organic matter (DOM) concentrations on the degradation kinetics were investigated. A mass of 97% minocycline was degraded in 45 min at 5% TiO_2_ dosage. The corresponding decomposition rate constant was 0.069 min^−1^. The inorganic anions affected the minocycline decomposition in the order of HCO_3_^−^ > Cl^−^ > SO_4_^2−^ > NO_3_^−^, which was confirmed by the results of electron spin resonance (ESR) spectra. The lowest electrical energy per order (*E_EO_*) was 6.5 Wh/L. Over five cycles, there was no change in the photocatalytic performance of the degrading minocycline. Those investigations suggested that effective degradation of minocycline could be reached in the PVDF–PVP–TiO_2_ fiber mats with a low energy consumption, good separation and, good recovery. Three photocatalytic decomposition pathways of minocycline were proposed: (i) hydroxyl substitution of the acylamino group; (ii) hydroxyl substitution of the amide group, and (iii) a cleavage of the methyl groups and further oxidation of the amino group by OH. Potential risks caused by TP159 and TP99 should not be ignored, while the TP90 are nontoxic. Tests indicated that the toxicity of the photocatalytic process may be persistent if minocycline and its products were not mineralized completely.

## 1. Introduction

The photocatalytic degradation of organic pollutants is a highly promising pathway toward a clean and low-energy consumption method of water treatment in the future [1,2,3,4,5,6,7]. The key issue for photocatalytic degradation of organic pollutants from water is the development of an efficient and stable photocatalyst without using sacrificial agents. Titanium dioxide (TiO_2_) has been one of the most extensively used semiconductor photocatalysts; it is biologically and chemically inert, non-toxic, and low cost [1,8,9,10,11]. In order to maximize the light adsorption and mass transfer rate, a suspended slurry of TiO_2_ nanoparticles is commonly used in the photocatalytic degradation of organic pollutants from water. However, despite that, in the in-situ production of reactive oxygen species (ROS) around TiO_2_ it is difficult to scavenge pollutants for the low adsorption capacity of TiO_2_ nanoparticles toward target contaminants [12,13]. Furthermore, an energy-intensive separation process was needed in the recovery of catalysts, such as the separation of titanium dioxide suspended slurry and membrane filtration [1]. Some substrates with a high adsorption capacity for objective containment can be used to immobilize TiO_2_ and enhance ROS reactions with the adsorbed pollution around TiO_2_ nanoparticles. At the same time, the costs of TiO_2_ nanoparticle separation and recycling have decreased. 

As reported earlier, TiO_2_ nanoparticles were blended with polymers to prepare fiber mats with improved practicability, adsorption capacity, and photocatalytic efficiency [1]. Appropriate polymers can effectively adsorb and bring preferential contaminants near photocatalytic sites for the more efficient use of short-lived ROS [1,14]. Polyvinylidene fluoride (PVDF) is a promising substrate for immobilizing TiO_2_, because it is stable enough to prevent the TiO_2_ from leaching, protects against ROS, and selectively adsorbs target pollutants, due to its strong C–F bonds and hydrophobic nature. Some investigators have fabricated PVDF with TiO_2_ fiber mats in order to solve the problem of catalyst recovery [1,15]. However, large doses of TiO_2_ were consumed through the photocatalysts being embedded inside the PVDF fiber. In order to improve the photocatalytic activity of PVDF–TiO_2_ fiber mats, water-soluble polyvinylpyrrolidone (PVP) was added to introduce a large number of pores and increase the exposure to the titanium dioxide. Therefore, PVDF–PVP–TiO_2_ membrane has an adsorption and photocatalytic activity that can solve the problem of catalyst recovery well and effectively removes organic pollutants from water.

Minocycline (7-dimethylamino-6-dimethyl-6-deoxytetracycline) is a broad-spectrum antimicrobial antibiotic. It can combine with tRNA to achieve a bacteriostatic effect. Minocycline has a wider antibacterial spectrum than similar drugs and has an antimicrobial activity. Its antimicrobial spectrum is similar to that of tetracycline, and it has a strong effect on Gram-positive bacteria, including tetracycline-resistant Staphylococcus aureus and Streptococcus, and the Gram-negative bacterium Neisseria gonorrhoeae. Hence, it is widely used in treating infections caused by bacteria. Because of the amine group (dimethylamine, DMA) in the structure of minocycline, N-nitrosodime-thylamine (NDMA) may be generated in water after treating with chloramine. Some investigators also revealed that NDMA can lead to a lifetime cancer risk level in humans and animals [16,17,18,19]. Minocycline at 4.9–8.2% (mol%) can be transformed to the NDMA precursor after a chloramine treatment [20,21]. Thus, minocycline was chosen as the target pollutant.

In this study, a PVDF–PVP–TiO_2_ adsorption–catalysis multifunctional membrane was fabricated by an electrospinning technology. The kinetics of PVDF–PVP–TiO_2_ fiber mats decomposing minocycline were studied. Effects of TiO_2_ dosage, minocycline concentration, inorganic anion, and pH value on the degradation kinetics were investigated. The photocatalytic byproduct was identified, and a possible degradation pathway was proposed for the photocatalytic degradation of minocycline. A quantitative structure–activity relationship (QSAR) model was adopted to predict the toxicities of minocycline and its degradation products. The energy consumption of photocatalytically degrading minocycline was estimated. This investigation may be helpful for constructing multifunctional composite electrospinning fiber films and recognizing the importance of recycling nanoparticle photocatalysts.

## 2. Materials and Methods

### 2.1. Materials

All chemical specifications are analytically pure. Titanium oxide, PVDF (MW = 400,000), acetone, methanol, formic acid, minocycline hydrochloride, PVP (MW = 58,000), and *N*,*N*-dimethylacetamide were all obtained from Aladdin Industrial Corporation, Shanghai, China, without further purification. Deionized (DI) water was produced by Milli-Q HX 7040.

### 2.2. Fabrication of Fiber Mats

To avoid the swathing of TiO_2_ nanoparticles in PVDF fiber mats, PVP (a water-soluble polymer) was added before electrospinning to promote pore formation (Lee et al., 2018). PVDF and PVP were dissolved in solvents contained *N*,*N*-Dimethylacetamide (DMAc) and acetone (6:5 *v*:*v*) along with 2%, 5%, and 10% TiO_2_ to prepare porous electrospun fibers. The weights of the TiO_2_ samples were 0.2, 0.5, and 1 g. The weight of the PVDF and PVP (PVDF:PVP = 2:1) was 10 g. The mixed solution was magnetically stirred for 18 h, then a 6 mL syringe filled with a homogenous polymer solution was connected to a needle. The conditions of the electrospinning were 15 kV voltage, 0.4 mL/h speed, and 15 cm distance between two parallel plates. The electrospinning process continued for 10 h under 25 °C and 60% relative humidity. After electrospinning, the PVP was washed out with DI water at 70 °C. Finally, the washed fiber mats were dried in a vacuum.

### 2.3. Characterization of Fiber Mats

The surface morphologies of the fiber mats were examined by field emission scanning electron microscopy (FE-SEM, Merlin, Jena, Germany) [22]. A transmission electron microscope (TEM, JEOL Ltd., Akishima, Japan) was operated at the electron accelerating voltage of 200 kV, the adhesive fibers on the copper wire were dried and observed in the electron microscope sample chamber [22]. An Alpha 300 M Raman spectrometer (WITec, Ulm, Germany), energy spectroscopy (EDS, Oxford, UK), and DSA100 (Hamburg, Germany) were used to gain insight into the microstructure and composition of fiber mats. Electron spin resonance (ESR) was used to detect and identify the radical intermediates with a spin trap compound (5,5-dimethyl-1-pyrroline *N*-oxide, DMPO).

### 2.4. Photocatalysis Experiments

Photodegradation experiments were performed under a xenon lamp (CEL-HXUV300H5, 300 W, Ceaulight, Beijing, China) radiating 365 nm UV (4 W). The fiber mats prepared were divided into 7 cm diameter circular (38.5 cm^2^) portions prior to photocatalysis experiments. The intensity received by the sample was 100 ± 5 mW/cm^2^. The distance from the light source to the specimen was 18 cm. Minocycline 10 mg/L solutions (200 mL) were irradiated in a special quartz reactor with 1 g fiber mats. The time of radiation was 60 min. The reusability of the electrospun membranes was estimated over five continuous cycles under 60 min of UV radiation.

### 2.5. Analytical Procedures

Minocycline concentrations were tested by ultra performance liquid chromatography (UPLC, Waters Acquity H-Class). The chromatographic column was BEH Phenyl, 2.1 mm × 50 mm, 1.7 μm; the temperature, 298 K; detection wavelength, 343 nm; mobile phase, methanol/formic acid (1%) = 1:9–4:6; and flow rate, 0.3 mL∙min^−1^. A UPLC/MS/MS system (Waters Acquity UPLC/Quattro Premier XE) was used to identify the degradation product of minocycline. The chromatographic column was BEH Phenyl 2.1 mm × 50 mm, 1.7 μm; the mobile phase, methanol/formic acid (0.1%) = 1:9–4:6; flow rate, 0.3 mL∙min^−1^; cone and capillary voltages (ESI), 3 V and 3 kV; mass spectrometry temperature, 673 K; and scanned mass, 50–500 a.m.u.

## 3. Results and Discussion

### 3.1. Characterization of Fiber Mats

**Figure 1:**
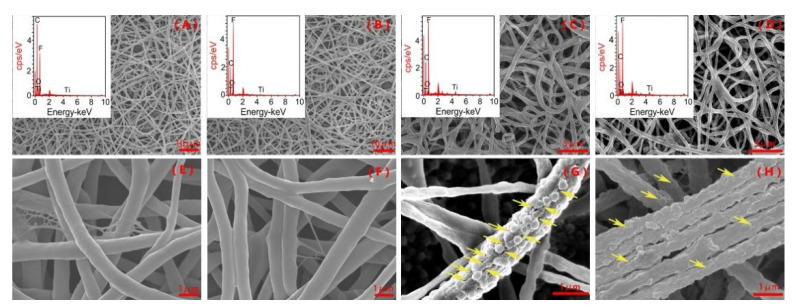
SEM images and EDS spectra of PVDF–PVP (**A**,**E**), PVDF–PVP–TiO_2_ 2% (**B**,**F**), PVDF–PVP–TiO_2_ 5% (**C**,**G**), and PVDF–PVP–TiO_2_ 10% (**D**,**H**) fiber mats. Arrows point to surface pores.

The morphology of PVDF–PVP and PVDF–PVP–TiO_2_ fiber mats was remarkably affected by the TiO_2_ dosage (Figure 1). The nonporous PVDF mats had a smooth surface (Figure 1A,E). The bead-like structures on this sample with 5% and 10% TiO_2_ contents likely indicate TiO_2_ aggregation (Figure 1G,H). The PVDF had a diameter of 214.80 ± 47.26 nm (Figure 1A,E). Adding TiO_2_ resulted in a rougher surface morphology (Figure 1G,H). PVDF–PVP–TiO_2_ 2%, PVDF–PVP–TiO_2_ 5%, and PVDF–PVP–TiO_2_ 10% had diameters of 256.37 ± 73.19 nm, 614.32 ± 139.26 nm, and 733.21 ± 183.43 nm, respectively. The most robust and TiO_2_-exposed mat was produced with PVDF–PVP–TiO_2_ 5%. 

**Figure 2:**
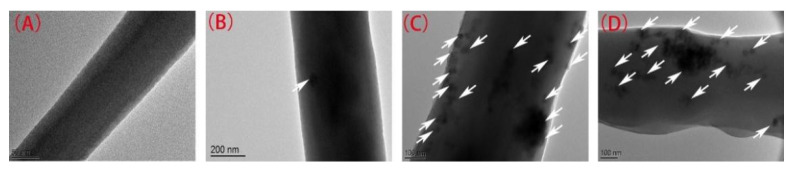
TEM images of PVDF–PVP (**A**), PVDF–PVP–TiO_2_ 2% (**B**), PVDF–PVP–TiO_2_ 5% (**C**), and PVDF–PVP–TiO_2_ 10% (**D**) fibers containing nanoscale (white arrows).

The results of the EDS spectra are illustrated in Figure 1 and listed in Table S1. As shown in Table S1, the combined weight percentages of Ti in PVDF–PVP–TiO_2_ 2%, PVDF-TiO_2_ 5%, and PVDF–PVP–TiO_2_ 10% fiber mats were 0.67%, 2.28%, and 3.82%, respectively. These weight percentages detected are lower than that of the added Ti. This may be due to the loss of mass in the fabrication process of the fiber mats and/or instrument error. The TEM results illustrated that TiO_2_ nanoparticles (Figure 2B–D) was present on the surface and in the interior of the fibers.

**Figure 3:**
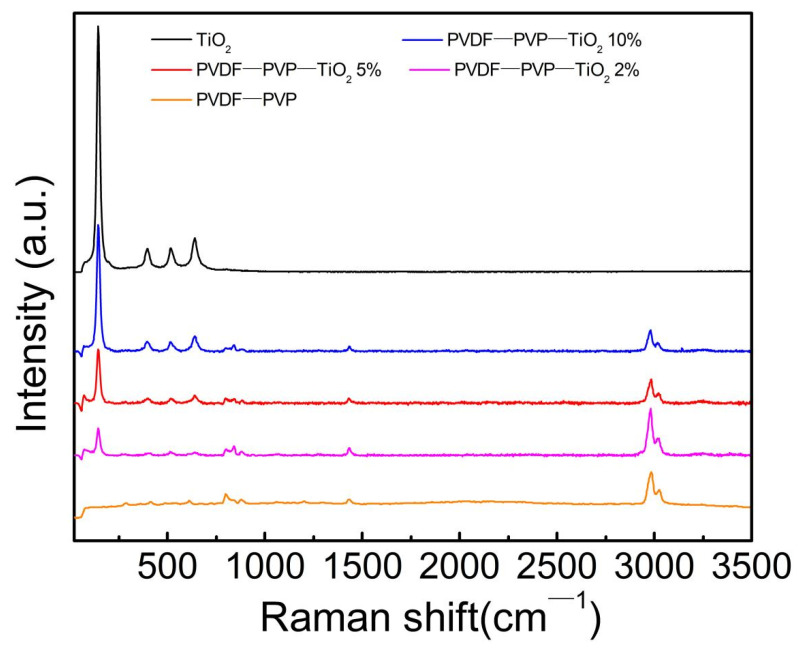
Raman spectroscopy of TiO_2_ nanoparticles, PVDF–PVP–TiO_2_ fiber mats, and PVDF–PVP.

The surface characteristics of TiO_2_ nanoparticles, PVDF–PVP–TiO_2_ fiber mats, and PVDF were further analyzed by Raman spectroscopy. Raman spectra showed that the spectral bands at 795 and 839 cm^−1^ were observed for both PVDF and PVDF–PVP–TiO_2_ fiber mats. In addition, higher bands at 2980 and 3024 cm^−1^ were also detected in the fiber mats (Figure 3), which may be associated with PVDF–PVP. The spectral bands at 143, 393, 515, and 638 cm^−1^ identifying TiO_2_ were more obvious with the increase of the TiO_2_ dosage in the PVDF–PVP–TiO_2_ fiber mats [23,24,25,26,27] (Figure 3).

### 3.2. Photocatalytic Activity of PVDF–PVP–TiO_2_ Fiber Mats

**Figure 4:**
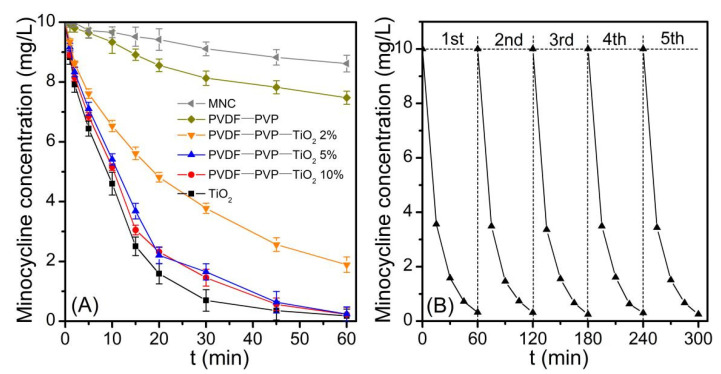
(**A**) Removal of minocycline ([minocycline] 0 = 10 mg/L) by adsorption and photocatalytic degradation under UV irradiation using mats made with PVDF–PVP, PVDF–PVP–TiO_2_ 2%, PVDF–PVP–TiO_2_ 5%, and PVDF–PVP–TiO_2_ 10%; (**B**) Reuse of PVDF–PVP–TiO_2_ 5% fiber mats over five cycles to remove minocycline ([minocycline] 0 = 10 mg/L) under similar irradiation conditions.

Photodegradation experiments (Figure 4) illustrated that there was a tardive decrease in the minocycline concentration under UV irradiation. The concentration of the minocycline during the treatment with PVDF fiber mats under UV irradiation also showed a slowly decreasing trend, but was a little faster than that with treatment by UV irradiation alone. This may indicate that the minocycline was adsorbed on the PVDF fiber mats, further suggested that PVDF can effectively adsorb minocycline. Methylene blue (MB) with similar properties is known to adsorb on hydrophobic surfaces such as PVDF [1,28,29]. The maximum minocycline adsorption capacity of PVDF–PVP–TiO_2_ 5% was 16.40 ± 0.45 mg g^−1^ based on a Langmuir isotherm analysis (Figure S1 and Table S2); these mats were ideal adsorption–catalysis multifunctional membranes.

**Figure 5:**
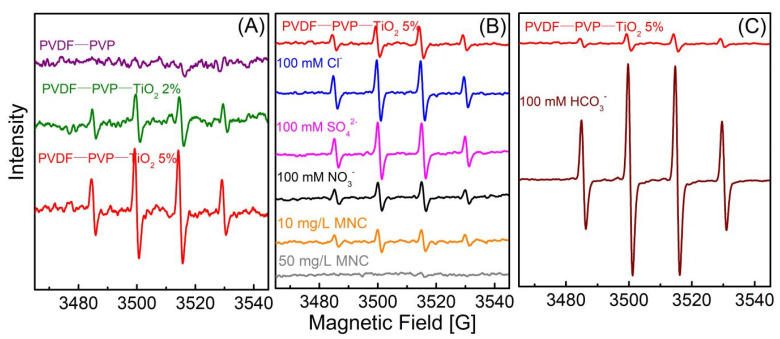
ESR spectra of (**A**) fiber mats (PVDF–PVP, PVDF–PVP–TiO_2_ 2%, and PVDF–PVP–TiO_2_ 5%) in DI water, (**B**) PVDF–PVP–TiO_2_ 5% with different solutions (DI water, 100 mM NaCl, 100 mM Na_2_SO_4_, 100 mM NaNO_3_, 10 mg/L minocycline (MNC), and 50 mg/L MNC), and (**C**) PVDF–PVP–TiO_2_ 5% with DI water and 100 mM NaHCO_3_.

Minocycline was effectively degraded by PVDF–PVP–TiO_2_ fiber mats and TiO_2_ under UV light (Figure 4A). The first-order rate constants (*k*) were 0.053, 0.069, 0.075, and 0.091 min^−1^ correspondingly (Table S3, Figure S2). The half-lives (*t*_1/2_) were 13.08, 10.05, 9.24, and 7.62 min correspondingly, which were similar to the trends of the *k* values. An almost complete degradation (>97%) of minocycline was achieved at 45 min using PVDF–PVP–TiO_2_ 5% fiber mats. This may be due to more OH being produced with a higher TiO_2_ dosage (Figure 5A) [30]. The *k* value was slightly enhanced when the percentage of TiO_2_ was greater than 5%. This phenomenon may be attributed to the following reason: the quantities of TiO_2_ nanoparticles exposed on the fibers slowly increased with the TiO_2_ dosage increase from 5% to 10%, which can be confirmed in the results of the SEM and TEM (Figure 1G,H and Figure 2C,D); hence, the excess TiO_2_ dosage decreased the photocatalytic performance of the fiber mats, and PVDF–PVP–TiO_2_ 5% fiber mats were chosen as the optimal photocatalyst in this study. In addition, over these five cycles there was no change in the photodegradation rate (Figure 4B). This may indicate that no or less minocycline and its intermediate products was retained on the surface of PVDF–PVP–TiO_2_ 5% fiber mats after five cycles.

### 3.3. Photocatalytic Degradation Kinetics of Minocycline

The influences of the concentration of minocycline, pH values, the concentrations of inorganic anions (Cl^−^, SO_4_^2−^, NO_3_^−^, and HCO_3_^−^), and DOM concentrations on the minocycline degradation using PVDF–PVP–TiO_2_ 5% fiber mats were researched. The photocatalytic degradation of minocycline followed pseudo-first-order kinetics (R^2^ > 0.97). The *k* and *t*_1/2_ were 0.006–0.121 min^−1^ and 5.71–115.52 min, respectively (Table S4). 

#### 3.3.1. Effect of Initial Concentration of Minocycline

Some investigations have reported that the concentration of pollutants is an important factor in water treatment [31]. Therefore, the influences of the minocycline concentration on the photocatalytic degradation rate were researched at 10–50 mg/L. The results are presented in Figure S3. The photocatalytic degradation rate of minocycline was significantly affected by the initial concentration. The degradation rate of decrease was more obvious at a lower initial concentration (Figure S3). At a higher initial concentration, the decomposition rate was lower as the order decreased. This indicated that the model of experimental data accorded with the Langmuir–Hinshelwood rate form. The *k* values were 0.069, 0.048, 0.045, and 0.041 min^−1^, and the corresponding *t*_1/2_ values were calculated to be 10.05, 14.50, 15.37, and 16.91 min at 10, 20, 30, and 50 mg/L, respectively. The *t*_1/2_ values increased and the *k* values decreased with the increase of initial concentration (Figure S3), while the initial rates exhibited an increased tendency of initial concentration in the inset of the figure (Figure S3). Those results may be due to the higher utilization of OH at higher initial concentrations [31]. This can also be confirmed by the results of an ESR spectra analysis, in which the spectral intensity of OH in 10 mg/L solutions was more obvious and stronger than that in 50 mg/L solutions (Figure 5B).

#### 3.3.2. Effect of Initial pH

Various pH values (3.0–11.0) had an obvious influence on the degradation of minocycline (Figure S4). The pH values were adjusted with 10 mM NaOH or 10 mM HCl to reach the desired pH values. There was no obvious degradation of minocycline after 60 min in darkness. However, within the pH range of 3.0–11.0, the degradation rate of minocycline significantly increased and ranged from 0.028 to 0.121 min^−1^. Under the conditions of alkalinity (pH = 11.0), the *k* values were four times higher than those under acidic (pH = 3.0) conditions. While for pH 5.0,7.0, 9.0, or 11.0, the *k* values of minocycline degradation were significantly enhanced and persisted (Figure S4, Table S3), which suggested that the photocatalytic oxidation may effectively degrade minocycline within a wide pH range. The high yield of OH was the main response to the high degradation rate at pH = 5.0–11.0 [1,32]. In addition, minocycline exists in five ionizable structures depending on the pH due to the dimethylamine and amide groups, and the pKa values were 2.63, 4.98, 7.86, and 10.05 respectively (Figure S5) [33,34]. Therefore, the initial pH may affect the initial dissociation species of minocycline and further impact its degradation kinetics. The rate of the minocycline degradation at pH 3.0–11.0 also confirmed those speculations. The same effects of the pH values on 2-chlorophenol degradation were reported in previous investigations [35,36].

#### 3.3.3. Effect of Inorganic Anions

Cl^−^, SO_4_^2−^, NO_3_^−^, and HCO_3_^−^ are ubiquitous anions in most wastewaters [37]. The influences of inorganic anions on the photocatalytic oxidation of minocycline were researched (Figure S5), and the results clearly illustrated that low NO_3_^−^ concentrations (10 and 25 mM) hardly affected the degradation of minocycline; however, high NO_3_^−^ concentrations (50–250 mM) showed slightly inhibiting influences (Figure S6). Cl^−^ and SO_4_^2−^ slightly enhanced the photocatalytic degradation of minocycline (Figure S6). The HCO_3_^−^ showed the greatest promotion influences on the minocycline degradation among those inorganic anions. Furthermore, the promotion influences of high HCO_3_^−^ concentrations (50–250 mM) were stronger than those of low HCO_3_^−^ (10–25 mM) concentrations (Figure S6). These results were confirmed by the ESR spectra analysis, in which the spectral intensity of OH with 100 mM HCO_3_^−^ was seven times stronger than that in DI water with no HCO_3_^−^ (Figure 5C). The changes in pH values caused by the HCO_3_^−^ may be responsible for the promoted degradation of minocycline solutions. Because the pH values of minocycline solutions (10 mg/L) were 4.58, 8.35, 8.43, 8.46, 8.39, and 8.35 with 0, 10, 25, 50, 100, and 250 mM HCO_3_^−^, respectively. The *k* values of minocycline degradation with HCO_3_^−^ remained in the range of 0.85 to 0.93 min^−1^, and were similar to the *k* value (0.093) at pH = 9.0. Similar promotion effects of the HCO_3_^−^/CO_3_^2−^ on oxytetracycline, oxcarbazepine, and DOM degradation were also found in many investigations [38,39,40,41], and they suggested that the promotion may be caused by the influence of a carbonate radical. The weaker spectral intensity of OH in 100 mM NO_3_^−^ also confirmed the inhibitory effect of NO_3_^−^ on minocycline degradation (Figure 5B). The slight promotion effect of Cl^−^, SO_4_^2−^ was also confirmed by the results of the ESR spectra (Figure 5B). Those inorganic anions affected the minocycline decomposition in the order of HCO_3_^−^ > Cl^−^ > SO_4_^2−^ > NO_3_^−^, and similar impacts of inorganic anions on the degradation of abacavir and bisphenol A were also reported by previous studies [37,39].

#### 3.3.4. Effect of DOM

DOM, here represented by humic acids (Fulvic acid ≥ 90%), is a heterogeneous mixture of organic compounds consisting of condensed aromatic hydrophobic cores surrounded by more hydrophilic molecules [41,42] and is a common composition excreted by microorganisms and breakdown products from decomposing organisms that is ubiquitous in natural water [37,43]. Hence, the influences of DOM on the photocatalytic degradation of minocycline were investigated, and the results are displayed in Figure S7 and Table S4. It was clear that the presence of DOM in the water inhibited the photocatalytic degradation of minocycline (Figure S7). The *k* values of minocycline degradation were 0.069, 0.039, 0.029, 0.025, and 0.021 min^−1^, and the corresponding *t*_1/2_ values were calculated to be 10.05, 17.73, 23.58, 27.84, and 32.85 min with 0, 10, 25, 50, and 100 mg/L DOM, respectively. The *t*_1/2_ values increased and the *k* values decreased with the increase of DOM concentration (Table S4). The inhibitory effect increased with higher DOM concentrations. Three main mechanisms of the inhibitory effect of DOM have been proposed: (i) DOM can scavenge OH [37,44,45]; (ii) DOM can act as a light screen and screens photo-chemically active light [46,47]; and (iii) DOM can react with contaminant intermediates [48,49]. 

Some investigators reported that effective photocatalytic degradation of DOM can be performed by using TiO_2_ under UV radiation [45]. This suggests that the first proposed mechanism may be genuine. As discussed in a previous investigation, the degradation of PhACs followed pseudo-first order kinetics, but the concentrations of PhACs did not exponentially decrease due to the screening effect of DOM [47]. In the present work, the photodegradation of minocycline followed pseudo-first order kinetics and exponentially decreased in time, which suggested that the second proposed mechanism might be negligible. Various organic moieties, such as phenolic function groups, are contained in DOM and can react with some oxidation intermediates [49]. Moreover, the concentrations of target pollutions directly decreased in the presence of DOM using Suwannee River fulvic acid as a reference DOM [48]. Therefore, the third proposed mechanism may be relevant to this study.

### 3.4. Degradation Mechanismand Toxicity Evaluation

**Figure 6:**
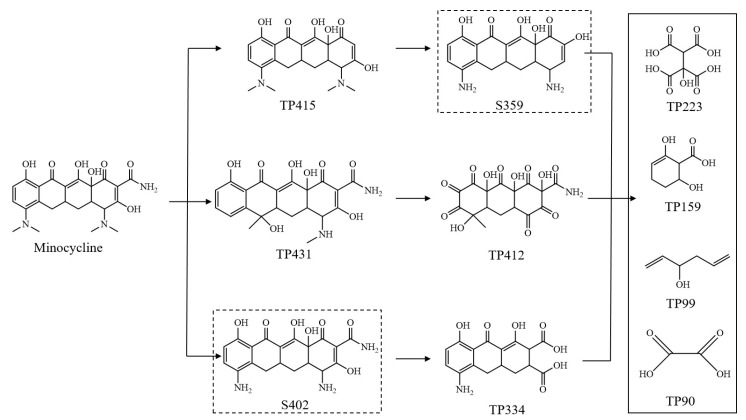
Possible photocatalysis pathways of minocycline degradation using PVDF–PVP–TiO_2_ 5% fiber mats. S402, S359 are not actually determined.

The degradation products of minocycline were examined by UPLC–MS/MS; the intermediates detected are listed in Table S5, and the possible degradation pathways are illustrated in Figure 6. TP431, TP415, TP412, TP334, TP,223, TP159, TP99, and TP90 were detected. Although the quantities of these products could not be exactly calculated due to the lack of commercial standards, the data could still be used to identify the reaction mechanism of minocycline oxidation by the attack of OH.

There may be three possible reaction mechanisms in the photocatalytic oxidation of minocycline with a PVDF–PVP–TiO_2_ membrane: (i) hydroxyl substitution of acylamino group; (ii) hydroxyl substitution of the amide group, and (iii) a cleavage of the methyl groups and further oxidation of the amino group by OH.

The attack of OH first leads to the cleavage of acylamino, dimethylamino, and methyl groups from minocycline generating TP415, TP431, S402, then further oxidation of the amino group and a cleavage of methyl groups by OH, which then leads to the formation of TP412, TP334, and S359. These are further degraded into TP223, TP 159, TP99, and TP90 by the oxidation of OH. Based on this mechanism, the priority sites for minocycline attacked by OH are the dimethylamine and amide functional groups, which is consistent with a previous investigation regarding the degradation of tetracycline using zero-valent iron and H_2_O_2_ [50,51].

The QSAR model embedded in the Ecological Structure Activity Relationships program is an effective method for predicting the toxicity of an organic pollutant and its degradation products. In this study, acute toxicity and chronic toxicity values (ChV) for minocycline and its degradation products were estimated, and these are listed in Table 1. The acute toxicity included LC50 (a compound concentration leading to 50% death of fish after 96 h and daphnids after 48 h) as well as EC50 (a compound concentration inhibiting the growth of green algae by 50% after 96 h). It was observed that minocycline showed acute and chronic toxicity, especially for daphnids and green algae. During the degradation of minocycline, some intermediates without toxicity (such as TP412, TP223, and TP90) were produced. It is worth noting that TP99 possessed very high toxicity to daphnids with LC50 and ChV values of 0.304 and 0.017 mg L^−1^, respectively. Besides TP99, TP159 also exhibited obvious toxicity to fish and daphnid. These results indicate that the toxicity of the photocatalytic process may persist if minocycline and its products are not mineralized completely. Therefore, the potential risks caused by minocycline and its degradation products should not be ignored considering the formation of TP99 and TP159.

### 3.5. Energy Consumption for Minocycline Photochemical Decomposition

Energy consumption plays an important role in practical applications [52,53], and therefore values for the electrical energy per order (EEO) at different conditions in this study were calculated and are displayed in Figure S8 and Table 1. The practicability of photocatalytic treatment is mainly determined by factors such as energy savings [13,54]. The EEO values were 6.2–36.4 Wh/L in the present study. Barring the acidic (pH = 3.0) and high DOM concentration (>25 mg/L) conditions, the EEO values were lower than 20 Wh/L. Low degradation rates of minocycline in acidic conditions and the scavenging of OH by DOM were responsible for the high EEO values. Generally, EEO values can be 0.1–100 Wh/L depending on pollutant and reactor configuration [55]. Therefore, our results clearly indicate that PVDF–PVP–TiO_2_ 5% fiber mats can efficiently decompose minocycline with a low energy consumption and that they were ideal adsorption–catalysis multifunctional membranes.

## 4. Conclusions

The photocatalytic degradation of minocycline was investigated via PVDF–PVP–TiO_2_ fiber mats under a UV–LED system. The results indicated that the optimal degradation rate constant was 0.069 min^−1^ at 5% TiO_2_ dosage, and the lowest EEO value was 6.5 Wh/L. NO_3_^−^ had a weakly inhibiting influence on the degradation of minocycline; Cl^−^ and SO_4_^2−^ slightly enhanced the photocatalytic decomposition of minocycline; HCO_3_^−^ promoted the generation of OH and enhanced the degradation. DOM in the water strongly inhibited the photocatalytic activity of PVDF–PVP–TiO_2_ 5% fiber mats for the scavenging of OH by the DOM. The results of a toxicity evaluation indicated that the toxicity of photocatalytic processes may persist if minocycline and its products are not mineralized completely. Potential risks caused by TP159 and TP99 should not be ignored, while the TP90 is nontoxic. These data support PVDF–PVP–TiO_2_ fiber mats as a promising method for degrading minocycline.

## Figures and Tables

**Table 1:** Toxicity evaluation of the intermediates of minocycline using a QSAR model.

Product	Acute Toxicity			Chronic Toxicity		
	(mg/L)			(*ChV*) (mg/L)		
	Fish (*LC*_50_)	Daphnid (*LC*_50_)	Green Algae (*EC*_50_)	Fish	Daphnid	Green Algae
Minocyline	1.78 × 10^2^	6.85 × 10^0^	9.14 × 10^1^	2.06 × 10^1^	8.04 × 10^0^	9.62 × 10^0^
TP431	4.94 × 10^2^	1.08 × 10^1^	2.97 × 10^2^	8.63 × 10^1^	2.64 × 10^1^	2.26 × 10^1^
TP415	6.35 × 10^1^	3.98 × 10^0^	2.86 × 10^1^	5.21 × 10^0^	2.50 × 10^0^	3.97 × 10^0^
TP412	6.41 × 10^6^	1.48 × 10^8^	4.86 × 10^4^	2.18 × 10^2^	1.06 × 10^5^	2.82 × 10^3^
TP334	7.75 × 10^2^	3.91 × 10^1^	3.71 × 10^1^	7.44 × 10^1^	3.25 × 10^1^	4.55 × 10^1^
TP223	1.01 × 10^8^	3.54 × 10^7^	3.51 × 10^6^	5.58 × 10^6^	8.88 × 10^5^	3.10 × 10^5^
TP159	5.84 × 10^1^	6.97 × 10^0^	2.92 × 10^3^	5.33 × 10^0^	7.26 × 10^−1^	2.91 × 10^2^
TP99	2.27 × 10^0^	3.04 × 10^−1^	2.95 × 10^1^	7.60 × 10 ^− 2^	1.70 × 10 ^− 2^	4.32 × 10^0^
TP90	1.68 × 10^5^	6.75 × 10^4^	1.21 × 10^4^	1.09 × 10^4^	2.52 × 10^3^	1.47 × 10^3^
S402	3.29 × 10^1^	3.24 × 10^0^	4.78 × 10^0^	1.68 × 10^1^	9.86 × 10^−1^	1.07 × 10^0^
S359	1.43 × 10^1^	1.67 × 10^0^	5.02 × 10^1^	1.54 × 10^0^	1.93 × 10^−1^	5.80 × 10^0^

*LC*_50_/*EC*_50_/*ChV* > 10^2^, not harmful, green label; 10^2^ > *LC*_50_/*EC*_50_/*ChV* > 10^1^, harmful, yellow label; 10^1^ > *LC*_50_/*EC*_50_/*ChV* > 10^0^, toxic, orange label; *LC*_50_/*EC*_50_/*ChV* < 10^0^, very toxic, red label.

## Data Availability

Not applicable.

## Supplementary Materials

The following are available online at https://www.mdpi.com/article/10.3390/ijerph182312339/s1, Figure S1: Isotherm model fitting for PVDF–PVP–TiO_2_ 5% fiber mats absorbing minocycline at 25 °C, Figure S2: The kinetics models for the photocatalytic degradation of minocycline using different photocatalyst, Figure S3: The effect of initial concentrations on the photocatalytic degradation rate of minocycline, Figure S4: The effect of initial pH values on the photocatalytic degradation rate of minocycline, Figure S5: Degradation rate constants (k) of the minocycline under the conditions with different Cl^−^, SO_4_^2−^, NO_3_^−^ and HCO_3_^−^ concentrations, Figure S6: Differently protonated forms of minocycline, Figure S7: The effect of DOM concentrations on the photocatalytic degradation rate of minocycline, Figure S8: EEO values at different DOM concentrations, initial pH, initial minocycline (MNC) concentration, SO_4_^2−^ concentrations, NO_3_^−^ concentrations, HCO_3_^−^ concentrations, and Cl^−^ concentrations, Table S1: Elemental composition and properties of the manufactured fiber mats, Table S2: The parameters of isotherm model for the adsorption of minocycline, Table S3: Kinetic parameters for the photocatalytic degradation of minocycline using different photocatalyst, Table S4: Kinetics for the photocatalytic degradation of minocycline at different initial conditions, Table S5: Identified degradation intermediates of minocycline in PVDF–PVP–TiO_2_ 5%/UV system, Table S6: The photocatalytic degradation conditions of minocycline in PVDF–PVP–TiO_2_ 5%/UV, and data analysis.
