# Supplementary material for: Degradation of Minocycline by the Adsorption–Catalysis Multifunctional PVDF–PVP–TiO2 Membrane: Degradation Kinetics, Photocatalytic Efficiency, and Toxicity of Products
Source: Int J Environ Res Public Health. 2021 Nov 24;18(23):12339. doi: 10.3390/ijerph182312339 (PMC8656511; doi:10.3390/ijerph182312339)
Supplement: Supplementary file 1 [file ijerph-18-12339-s001.zip › ijerph-1458892-supplementary.pdf]

Degradation of Minocycline by the Adsorption–Catalysis Multifunctional PVDF–PVP–TiO_2 Membrane: Degradation Kinetics, Photocatalytic Efficiency, and Toxicity of Products

Chengzhi Zhou[1], Yanlong Sun[2], Fan Zhang[3], Yuandong Wu[4*]

[1] Qingdao Engineering Research Center for Rural Environment, College of Resource and Environment, Qingdao Agricultural University, Qingdao 266109, China; zhoucz@qau.edu.cn
[2] College of Geography and Environmental Sciences, Zhejiang Normal University, Jinhua 321004, China; sunyanlong@zjnu.edu.cn
[3] School of Electrical and Information Technology, Yunnan Minzu University, Kunming 650031, China; zhangfan2819@163.com
[4] Shenzhen Institute, Peking University, Shenzhen 51800, China
* Correspondence: wuyd@ier.org.cn

Yuandong Wu, *wuyd@ier.org.cn*

Chengzhi Zhou, *zhoucz@qau.edu.cn*

Yanlong Sun, *sunyanlong@zjnu.edu.cn*

Fan Zhang，zhangfan2819@163.com

Number of pages: 14

Number of Figures: 7

Number of Tables: 7

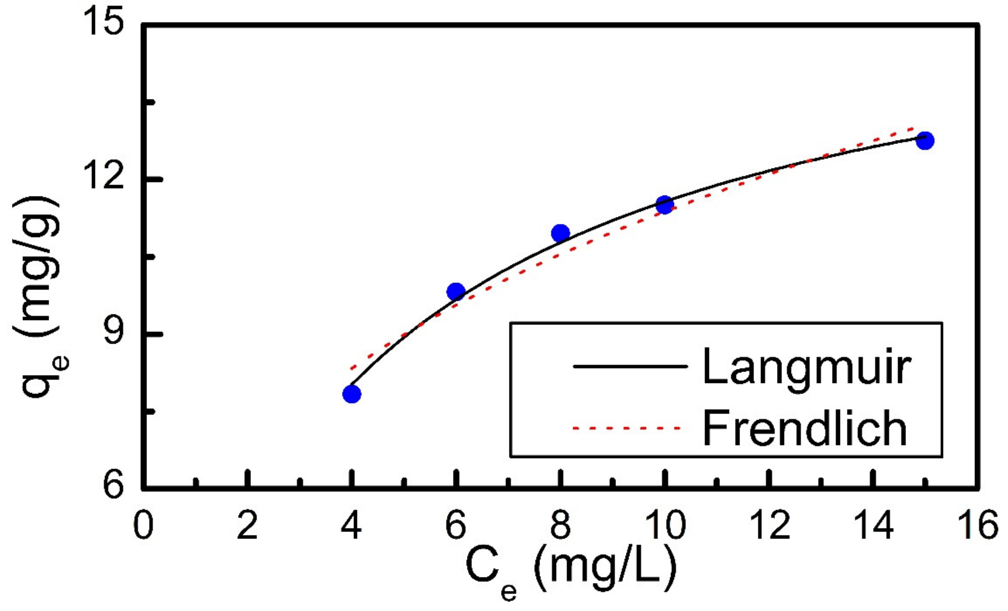

Figure S1. Isotherm model fitting for PVDF-TiO_2 5% fiber mats absorbing minocycline at 25 °C.

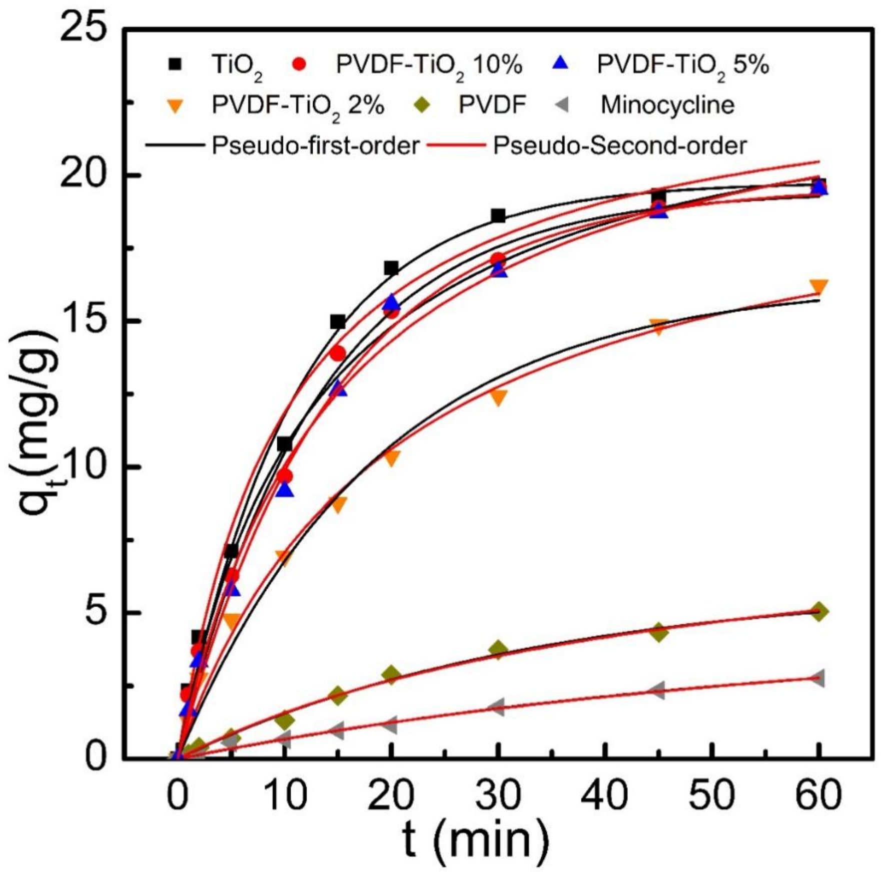

Figure S2. The kinetics models for the photocatalytic degradation of minocycline using different photocatalyst.

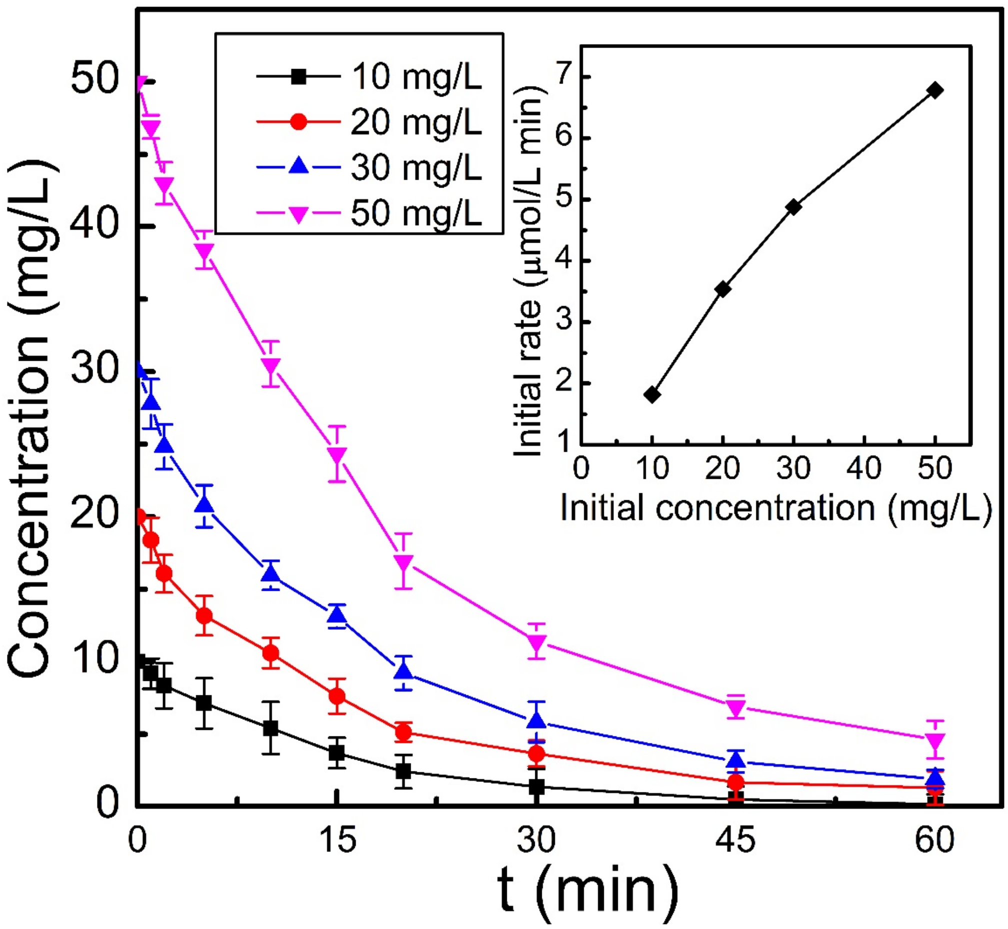

Figure S3. The effect of initial concentrations on the photocatalytic degradation rate of minocycline

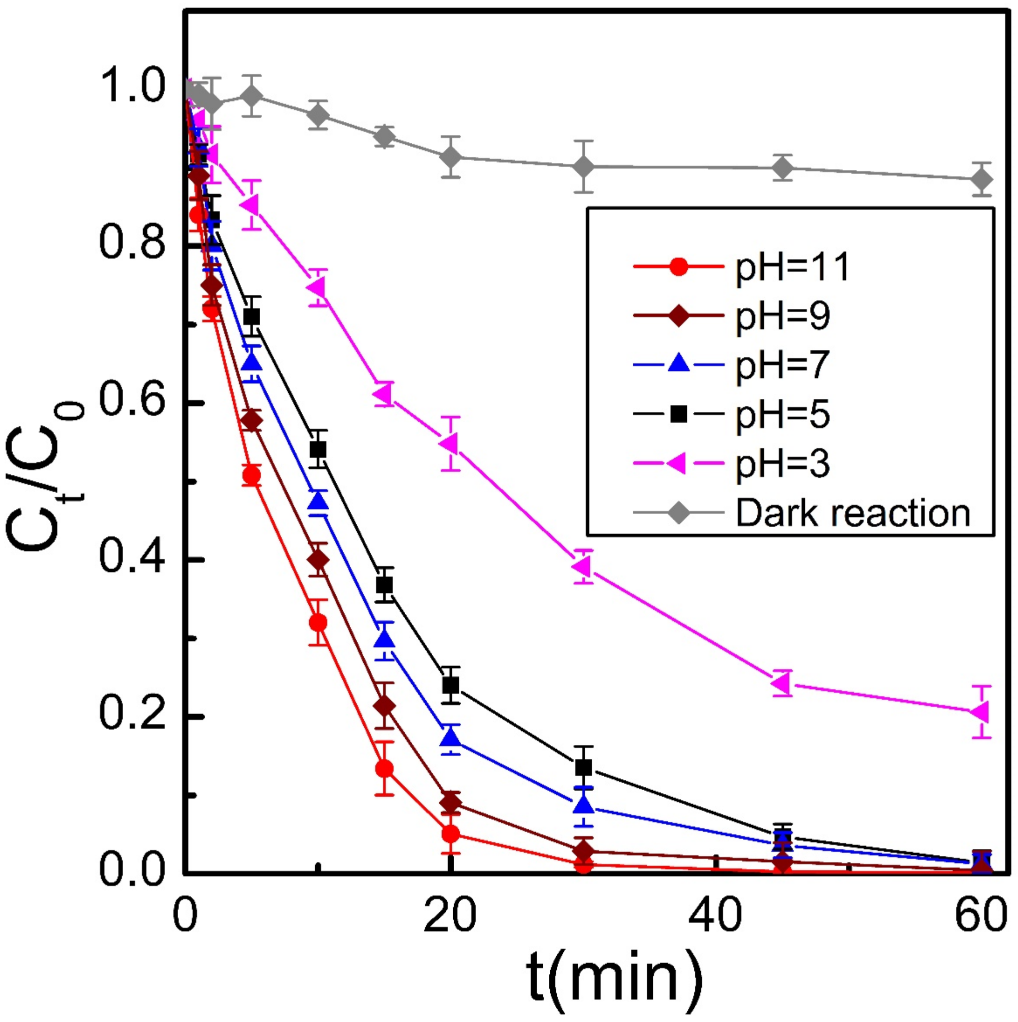

Figure S4. The effect of initial pH values on the photocatalytic degradation rate of minocycline. C_0 and C_t are the initial and time *t* (min) concentrations of minocycline, respectively.

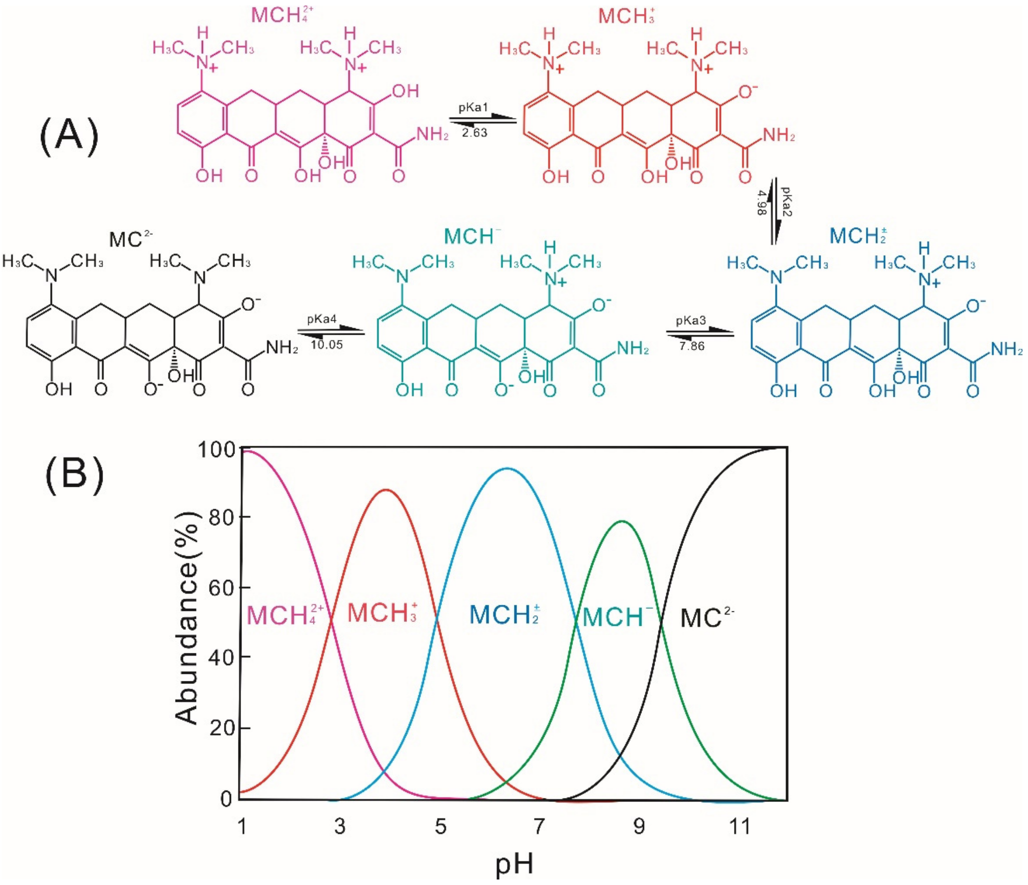

Figure S5. Differently protonated forms of minocycline

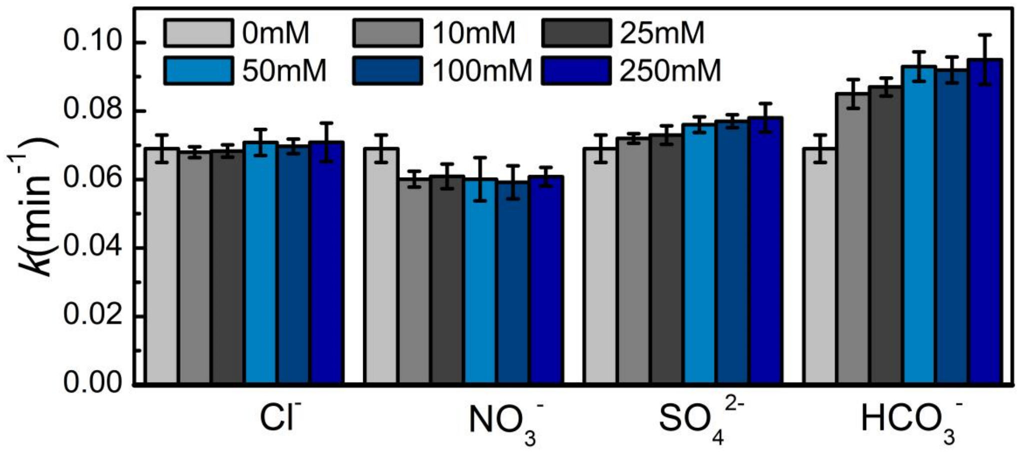

Figure S6. The influences of inorganic anions on the photocatalytic oxidation of minocycline.

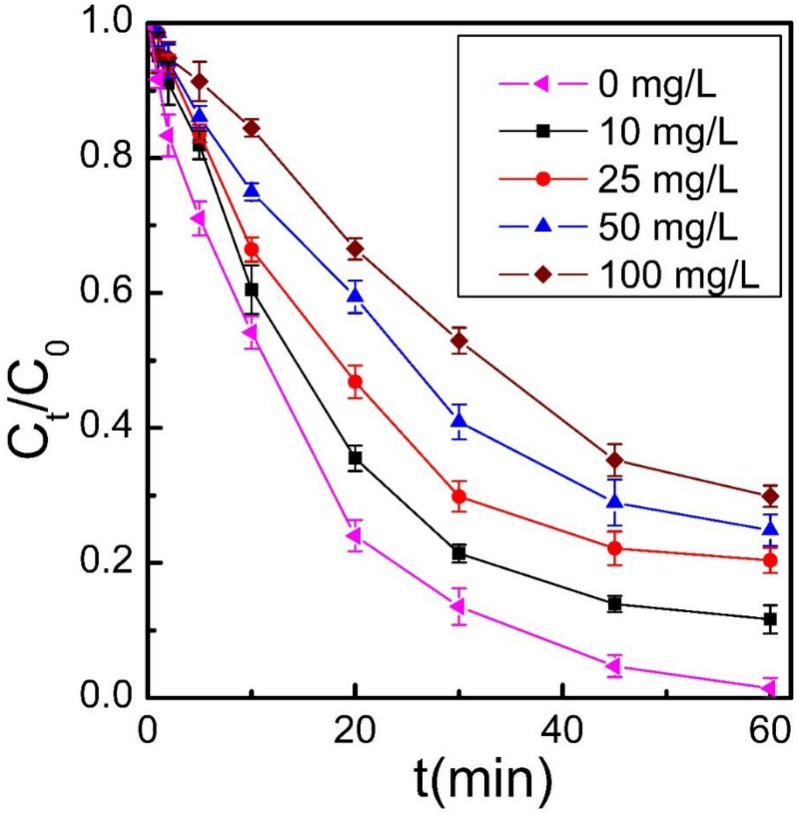

Figure S7. The effect of DOM concentrations on the photocatalytic degradation rate of minocycline. C_0 and C_t are the initial and time *t* (min) concentrations of minocycline, respectively.

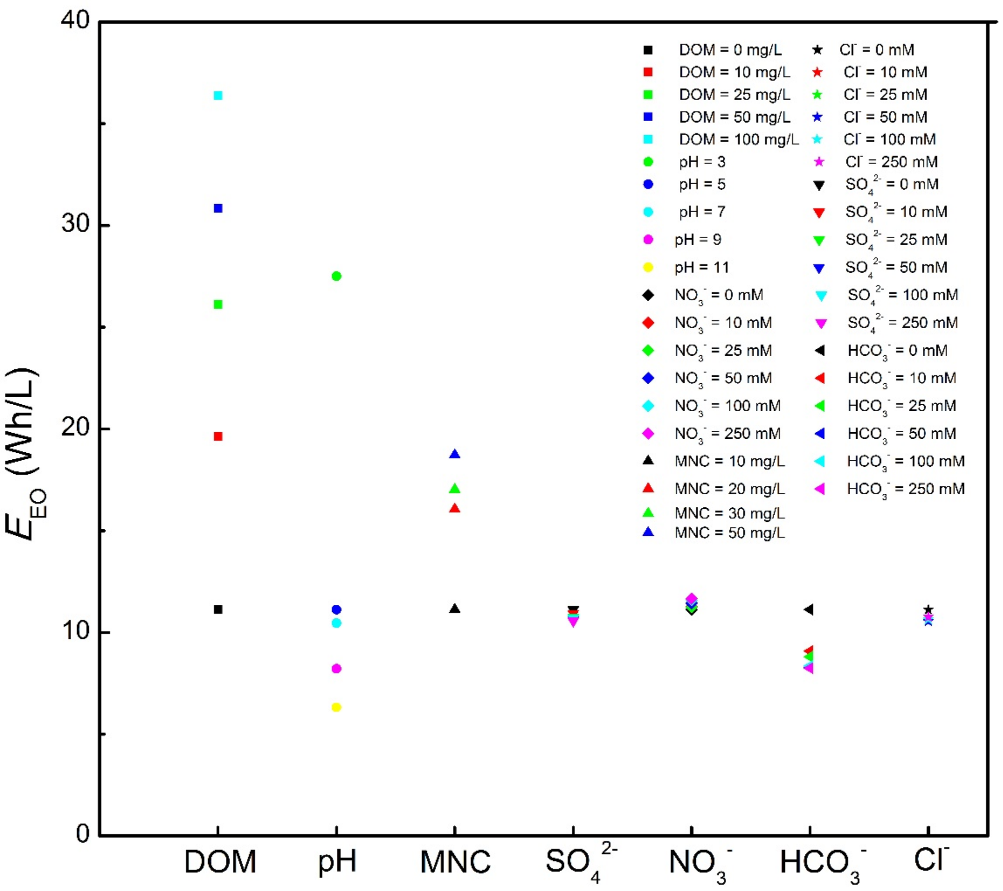

Figure S8. E_{EO} values at different DOM concentrations, initial pH, initial minocycline (MNC) concentration, SO_4^{2-} concentrations, NO_3^- concentrations, HCO_3^- concentrations, and Cl^- concentrations

Adsorption kinetic models:

Adsorption kinetic experiments are conducted in 25 mg/L minocycline solutions with 0.5 g adsorbent at 25 °C. Pseudo-first-order model, pseudo-second order model and intra-particle diffusion model are applied to interpret the experimental data.

Three different models were used to fit the adsorption kinetics. The pseudo-first-order model, pseudo-second-order model and intra-particle model, which can be expressed as Equation 1, 2, and 3.

Pseudo-first-order model:

$$ln\left(1-\frac{q_t}{q_e}\right)=-k_1 t \qquad (1)$$

Pseudo-second-order model:

$$\frac{t}{q_t} = \frac{1}{k_2 q_e^2} + \frac{t}{q_e} \qquad (2)$$

Intra-particle diffusion model:

$$\mathrm{q_t} = \mathrm{k_3 t^{1/2}} \qquad (3)$$

where q_t (mg·g^{-1}) and q_e (mg·g^{-1}) are the amount of minocycline degraded at any time t (min) and at equilibrium, respectively; k (g·mg^{-1} min^{-1}) represents the rate constant of each model; and C (mg·g^{-1}) is the boundary layer thickness. The fitting results of the pseudo-first-order model and the pseudo-second-order model are shown in follow.

Procedures for determination of the isotherms

The adsorption experiments are performed at 25 °C, and the minocycline concentration is range from 4, 6, 8, 10, and 15 mg/L. In order to describe the interactive behavior between solute and adsorbent, Langmuir (Equation 4) and Freundlich (Equation 5) models are applied to analyze the adsorption data.

The adsorption capacity of PVDF-PVP -TiO_2 5% were evaluated at different concentration of minocycline. The equilibrium concentrations were used for the following Langmuir (Eq. 4) and Freundlich (Eq. 5) isotherm model analysis.

$$q_e = \frac{Q_m K_L C_e}{1 + K_L C_e} \qquad (4)$$

$$q_e = K_F C_e^{\frac{1}{n}} \qquad (5)$$

where q_e and C_e are the amount of minocycline adsorbed on the PVDF-PVP-TiO_2 5% fiber mats and minocycline concentration in the aqueous solution at equilibrium, respectively. Q_m is the maximum adsorption capacity, K_L is the Langmuir constant related to the binding site affinity, K_F is the Freundlich constant related to the adsorption capacity, and 1/n is the Freundlich constant related to the adsorption intensity. As shown in Figure SI 1, the

equilibrium adsorption data were better fitted by Langmuir isotherm that assumes uniform monlolayer adsorption. The isotherm model parameters were represented in Table S2.

Electrical energy per order

Electrical energy per order (E_{EO}), defined as the number of kilowatts-hour (kWh) of electrical energy required to remove the concentration of a pollutant (e.g., BPA, EE2, MB) by 1 order of magnitude (90%) in one cubic meter of contaminated water, was calculated as follows Eq. 6[1-3]:

$$E_{\mathrm{EO}}(\mathrm{kWh}/L/\mathrm{order}) = \frac{E \times \left(\frac{t}{60}\right)}{V \times \log\left(\frac{C_0}{C}\right)} \quad (6)$$

where *E* is defined here as the irradiance delivered from the light sources (kWh), *t* is irradiation (or operation) time (min), V is solution volume (L), C_0 and C are the initial and time *t* (min) concentrations of pollutant, respectively. This equation can be simplified by using pseudo first-order rate constant (k, min^{-1}) as Eq. 7:

$$E_{\mathrm{EO}}(\mathrm{kWh}/L/\mathrm{order}) = \frac{E}{V \times 60 \times 0.4343 \times k} \quad (7)$$

The *k* values with their respective standard errors were estimated by fitting the data to first order kinetics.

Table S1 Elemental composition and properties of the manufactured fiber mats.

Parameter	PVDF-PVP	PVDF-PVP TiO_2 2%	PVDF-PVP TiO_2 5%	PVDF-PVP TiO_2 10%
Elemental composition% (C/F/Ti/O)[a]	46.52/53.24.00/0/0.23	41.73/55.36/0.67/2.24	41.38/51.38/2.28/4.95	40.26/50.63/3.82/5.29
Mean fiber dia[b]	214.80±47.26	256.37±73.19	614.32±139.26	733.21±183.43
Water contact angles	106.0 ± 1.0°	91.3± 0.9°	88.2± 3.3°	90.4± 2.2°

[a]EDS (wt%). [b]Based on SEM images

Table S2: The parameters of isotherm model for the adsorption of minocycline

Sample	Langmuir			Freundlich		
	K_L（L/mg）	R^2	Q_m（mg/g）	K_F（L/g)	R^2	1/n
PVDF-TiO_2 5%	0.240±0.020	0.990	16.40±0.454	5.506±0.500	0.943	0.340±0.043

Table S3: Kinetic parameters for the photocatalytic degradation of minocycline using different photocatalyst

Adsorbent	Pseudo-first-order models			Pseudo-second-order models		
	q_e (mg·g^{-1})	k_1 (min^{-1})	R^2	q_e (mg·g^{-1})	k_2 (min^{-1})	R^2
Minocycline	4.332±0.560	0.017±0.003	0.992	7.154±1.142	0.001±0.000	0.991
PVDF	6.050±0.319	0.030±0.003	0.995	9.163±0.758	0.002±0.000	0.993
PVDF-TiO_2 2%	16.375±0.685	0.053±0.005	0.987	21.393±0.872	0.002±0.000	0.994
PVDF-TiO_2 5%	19.644±0.438	0.069±0.004	0.995	24.847±0.0973	0.003±0.000	0.993
PVDF-TiO_2 10%	19.457±0.437	0.077±0.005	0.935	24.108±0.823	0.003±0.000	0.994
TiO_2	19.767±0.379	0.091±0.005	0.995	23.929±0.922	0.004±0.000	0.990

Table S4: Kinetics for the photocatalytic degradation of minocycline at different initial conditions

Initial conditions		Rate constants	Half-Lives	R^2
Minocycline concentration (mg/L)	10	0.069	10.05	0.995
	20	0.0478	14.50	0.973
	30	0.0451	15.37	0.982
	50	0.041	16.91	0.984
pH value	Dark	0.002	346.58	0.848
	3	0.0279	24.84	0.986
	5	0.069	10.05	0.995
	7	0.0734	9.44	0.994
	9	0.0934	7.42	0.980
	11	0.1214	5.71	0.978
Cl^- concentration (mM)	0	0.069	10.05	0.995
	10	0.0721	9.61	0.993
	25	0.0722	9.60	0.989
	50	0.0728	9.52	0.995
	100	0.0721	9.61	0.993
	250	0.0713	9.72	0.993
SO_4^{2-} concentration (mM)	0	0.069	10.05	0.995
	10	0.0702	9.87	0.986
	25	0.0713	9.72	0.983
	50	0.0721	9.61	0.990
	100	0.0718	9.65	0.991
	250	0.0726	9.55	0.993
NO_3^- concentration (mM)	0	0.069	10.05	0.995
	10	0.0681	10.18	0.999
	25	0.0683	10.15	0.999
	50	0.0671	10.33	0.997
	100	0.0662	10.47	0.998
	250	0.0658	10.53	0.998
HCO_3^- concentration (mM)	0	0.069	10.05	0.995
	10	0.0845	8.20	0.990
	25	0.0872	7.95	0.970
	50	0.0926	7.49	0.983
	100	0.0919	7.54	0.982
	250	0.0931	7.45	0.983
DOM	0	0.069	10.05	0.995
	10	0.0391	17.73	0.972
	25	0.0294	23.58	0.971
	50	0.0249	27.84	0.982
	100	0.0211	32.85	0.991

Table S5. Identified degradation intermediates of minocycline in PVDF-TiO_2 5% / UV system.

Product	$[M+H]^+$	Proposed Formula	Structure
Minocycline	458.5	$C_{23}H_{28}N_3O_7^+$	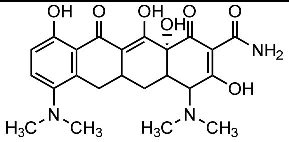
TP431	431.1		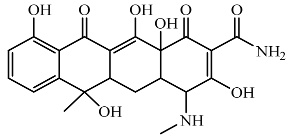
TP415	415.2	$C_{22}H_{27}N_2O_6^+$	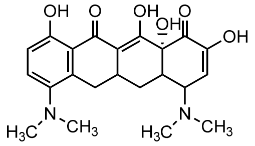
TP412	412.2		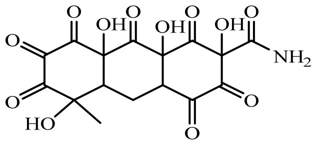
TP334	334.1	$C_{16}H_{16}NO_7^+$	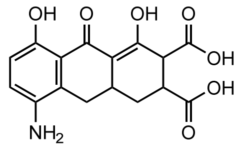
TP223	223.1		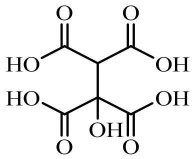
TP159	159.2	$C_7H_{11}O_4^+$	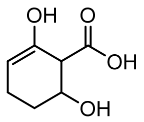
TP99	99.1	$C_6H_{10}O^+$	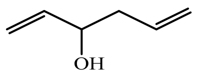
TP90	90.2	$C_2H_3O_4^+$	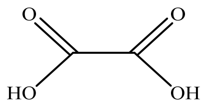

Table S6. The photocatalytic degradation conditions of minocycline in PVDF-TiO_2 5%/UV

target compound	catalyst	substrate	light source	loading	initial target concentration	removal rate
**Minocycline**	TiO_2	PVDF	λ = 365 nm (4 W)	38.5 cm^3/200 ml	21.8μM	0.069min^{-1}

References

1. Lanzarini-Lopes, M., Garcia-Segura, S., Hristovski, K., Westerhoff, P., 2017. Electrical energy per order and current efficiency for electrochemical oxidation of p-chlorobenzoic acid with boron-doped diamond anode. Chemosphere, 188, 304-311.
2. Daneshvar, N., Aleboyeh, A., Khataee, A. R., 2005. The evaluation of electrical energy per order (EEo) for photooxidative decolorization of four textile dye solutions by the kinetic model. Chemosphere, 59, 761-767.
3. Lee, C. G., Javed, H., Zhang, D., Kim, J. H., Westerhoff, P., Li, Q., Alvarez, P. J., 2018. Porous electrospun fibers embedding TiO_2 for adsorption and photocatalytic degradation of water pollutants. Environ. Sci. Technol. *52*, 4285-4293.
